# Plant Cell Wall Proteins: A Large Body of Data, but What about Runaways?

**DOI:** 10.3390/proteomes2020224

**Published:** 2014-04-17

**Authors:** Cécile Albenne, Hervé Canut, Laurent Hoffmann, Elisabeth Jamet

**Affiliations:** 1 Université de Toulouse; UPS, UMR 5546, Laboratoire de Recherche en Sciences Végétales, BP 42617 Auzeville, F-31326 Castanet-Tolosan, France; E-Mails: albenne@lrsv.ups-tlse.fr (C.A.); canut@lrsv.ups-tlse.fr (H.C.); hoffmann@lrsv.ups-tlse.fr (L.H.); 2 CNRS, UMR 5546, BP 42617, F-31326 Castanet-Tolosan, France

**Keywords:** cell wall, plant, proteomics

## Abstract

Plant cell wall proteomics has been a very dynamic field of research for about fifteen years. A full range of strategies has been proposed to increase the number of identified proteins and to characterize their post-translational modifications. The protocols are still improving to enlarge the coverage of cell wall proteomes. Comparisons between these proteomes have been done based on various working strategies or different physiological stages. In this review, two points are highlighted. The first point is related to data analysis with an overview of the cell wall proteomes already described. A large body of data is now available with the description of cell wall proteomes of seventeen plant species. CWP contents exhibit particularities in relation to the major differences in cell wall composition and structure between these plants and between plant organs. The second point is related to methodology and concerns the present limitations of the coverage of cell wall proteomes. Because of the variety of cell wall structures and of the diversity of protein/polysaccharide and protein/protein interactions in cell walls, some CWPs can be missing either because they are washed out during the purification of cell walls or because they are covalently linked to cell wall components.

## 1. Introduction

Plant cell wall proteomics is a tricky field of research, since proteins are not only minor components of plant cell walls, but are also trapped in complex networks of polysaccharides with which they can interact. Plant cell walls are mainly composed of cellulose microfibrils wrapped in and connected with hemicelluloses and inserted into a complex pectin gel [1]. At the end of growth, secondary walls are formed [2]. Such walls are more rigid and may contain lignin. The structure and composition of cell walls are constantly modified to allow plant growth and development, and to contribute to the adaptation of plants to their changing environment [3,4,5]. All these processes involve *de novo* assembly and/or remodeling of wall components as well as signaling processes [6]. 

Cell wall proteins (CWPs) are the “blue collar workers,” modifying cell wall components and customizing them to confer appropriate properties to cell walls [6]. They also contribute to signaling by interacting with plasma membrane receptors or by releasing signal molecules such as peptides or oligosaccharides [7,8,9]. Thus, a large variety of proteins are present in cell walls [10]. They have different physico-chemical properties, they may interact with other cell wall components and their relative abundance is variable. Proteomics strategies should allow the full inventory of proteins in a tissue, an organ or an organelle at a given stage of development or in response to an external stimulus. However, in the case of cell walls, these strategies are particularly difficult to establish [11]. The three main drawbacks are: (i) cell walls constitute open compartments, (ii) proteins are trapped in a complex polysaccharide matrix with which they interact and (iii) most CWPs are modified at the post-translational level. Two types of flowcharts have been designed and used: non-destructive or non-disruptive ones elute proteins outside the cells without disrupting plasma membranes; destructive or disruptive ones start with the purification of cell walls followed by the elution of proteins with various solutions. Each of them has advantages and drawbacks which have been previously reviewed [10,12]. The combination of these strategies has led to the identification of hundreds of proteins in various plants and in different organs. *Arabidopsis thaliana* has been the most studied plant with 500 CWPs identified at present, representing about one fourth of the expected CWPs. In *Oryza sativa* and *Brachypodium distachyon*, the second and third most studied plants, 314 and 270 CWPs have been identified so far respectively. 

Comparisons between different cell wall proteomes have been done using two criteria. In a few cases, different strategies have been used to analyze the same organs. For example, *Populus deltoides* CWPs have been identified either after separation by 1D-electrophoresis followed by LC-MS/MS analysis or after direct analysis by LC-MS/MS [13]. Two partly overlapping sets of proteins have been identified showing that different technologies are required to enlarge the coverage of cell wall proteomes. In other cases, organs at different stages of development or different organs have been analyzed using the same strategies. Cell wall proteomes of *A. thaliana* etiolated hypocotyls have been analyzed 5 or 11 days after germination [14]. In the same way, cell wall proteomes have been studied in growing and mature leaf and stems of *B. distachyon* [15], and in apical and basal stems of *Medicago sativa* [16]. Such experiments have allowed the identification of candidate proteins possibly involved in cell wall extension or in cell wall strengthening at the end of growth. Finally, a quantitative approach has allowed the identification of the *A. thaliana* GLIP1 GDSL lipase as a contributor to plant defense against *A. brassicicola* infection [17].

Despite the accumulation of data, well-known CWPs are still under-represented in cell wall proteomes, like structural proteins forming covalent networks, *i.e*., Proline-Rich Proteins (PRPs) and extensins (EXTs), or highly glycosylated proteins, like ArabinoGalactan Proteins (AGPs). In addition, the analysis of the content of the buffers used during the washings steps of cell walls during their purification has shown that some proteins are lost at that step. In this review, we focus on two points: (i) an overview of the existing cell wall proteomics data highlighting differences between monocots and dicots in relation to differences in cell wall composition and structure or between cell wall proteomes of different organs and (ii) the limitations to the full coverage of cell wall proteomes.

## 2. A Large Body of Data

With 53 papers reporting plant cell wall proteomes, much data has been accumulated during the last 15 years (Table 1). Seventeen plant species have been the subject of investigations among which 13 dicots and 4 monocots. As previously reviewed, different plant organs, mainly roots, hypocotyls, stems, leaves, ovules and fruits, as well as suspension cultures and seedlings grown in liquid medium have been studied using different strategies [10,18]. Xylem sap proteomes have been considered in this analysis because they contain many secreted proteins which could originate from root stele cells or from dying xylem cells [19]. Altogether, 2170 CWPs encoded by distinct genes have been identified. Classifications into functional classes have been proposed to get overviews of cell wall proteomes [10,20]. It is noteworthy that the same classes have been found in all proteomes: proteins acting on polysaccharides (PAC, e.g., glycoside hydrolases, carbohydrate esterases and lyases, expansins), oxido-reductases (OR, e.g., peroxidases, multicopper oxidases, blue copper binding proteins and multicopper oxidases), proteases (P, e.g., Asp proteases, Cys proteases, Ser proteases, Ser carboxypeptidases), proteins having interacting domains (ID) with polysaccharides (e.g., lectins) or proteins (e.g., enzyme inhibitors, leucine-rich repeats proteins), proteins possibly involved in lipid metabolism (LM, e.g., lipases GDSL, lipid transfer proteins), proteins possibly involved in signaling (S, e.g., arabinogalactan proteins), structural proteins (SP, e.g., leucine-rich repeat extensins, glycine-rich proteins) and proteins of yet unknown function (UF). Proteins with predicted function which are not falling into these categories have been grouped into the miscellaneous class (M, e.g., purple acid phosphatases, phosphate-inducible (phi) proteins, germin and germin-like proteins). 

**Table 1:** Plant cell wall proteomics (CWPs) studies.

Plant species	Type of proteome	Number of identified CWPs ^a^	References
Dicots			
*Arabidopsis thaliana*	cell wall	913	[14,17,21,22,23,24,25,26,27,28,29,30,31,32,33,34,35,36]
	*N*-glycoproteome	200	[37,38]
		**495**	
*Brassica napus/oleracea*	xylem sap	147	[19,39]
	*N*-glycoproteome	92	[19]
		**162**	
*Cicer arietinum*	cell wall	nd	[40,41,42]
*Glycine max*	cell wall	nd	[43]
*Gossypium hirsutum*	*N*-glycoproteome	**116**	[44]
*Helianthus annuus*	cell wall	nd	[45]
*Linum usitatissimum*	cell wall	**106**	[46]
*Medicago sativa*	cell wall	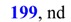	[16,47]
*Nicotiana benthamiana*	cell wall	nd	[48]
*Nicotiana tabacum*	cell wall	nd	[34,49,50,51]
*Populus deltoides *	cell wall	144	[13]
*P. trichocarpa x P. deltoides *(hybrid poplar)	xylem sap	33	[52]
		**142**	
*Solanum lycopersicum*	cell wall	nd, 60	[34,53]
	*N*-glycoproteome	104	[20]
		**161**	
*Solanum tuberosum*	cell wall	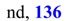	[54,55]
Monocots			
*Brachypodium distachyon*	cell wall	689	[15]
		**314**	
*Oryza sativa*	cell wall	381	[56,57,58,59,60]
		**270**	
*Saccharum officinarum*	cell wall	**69**	[61]
*Zea mays*	cell wall,	nd	[62,63]
	xylem sap	nd	[64]

^a^ All these proteomes are in the WallProtDB database (See Supplementary Material). Only proteins having a predicted signal peptide are considered (see Supplementary Material). The number of identified proteins is only mentioned when the identification has been done using homologous sequences. Otherwise, nd means that this number could not be calculated. Numbers in black correspond to the total number of proteins identified whereas numbers in bold blue correspond to numbers of different proteins identified in each species.

**Figure 1:**
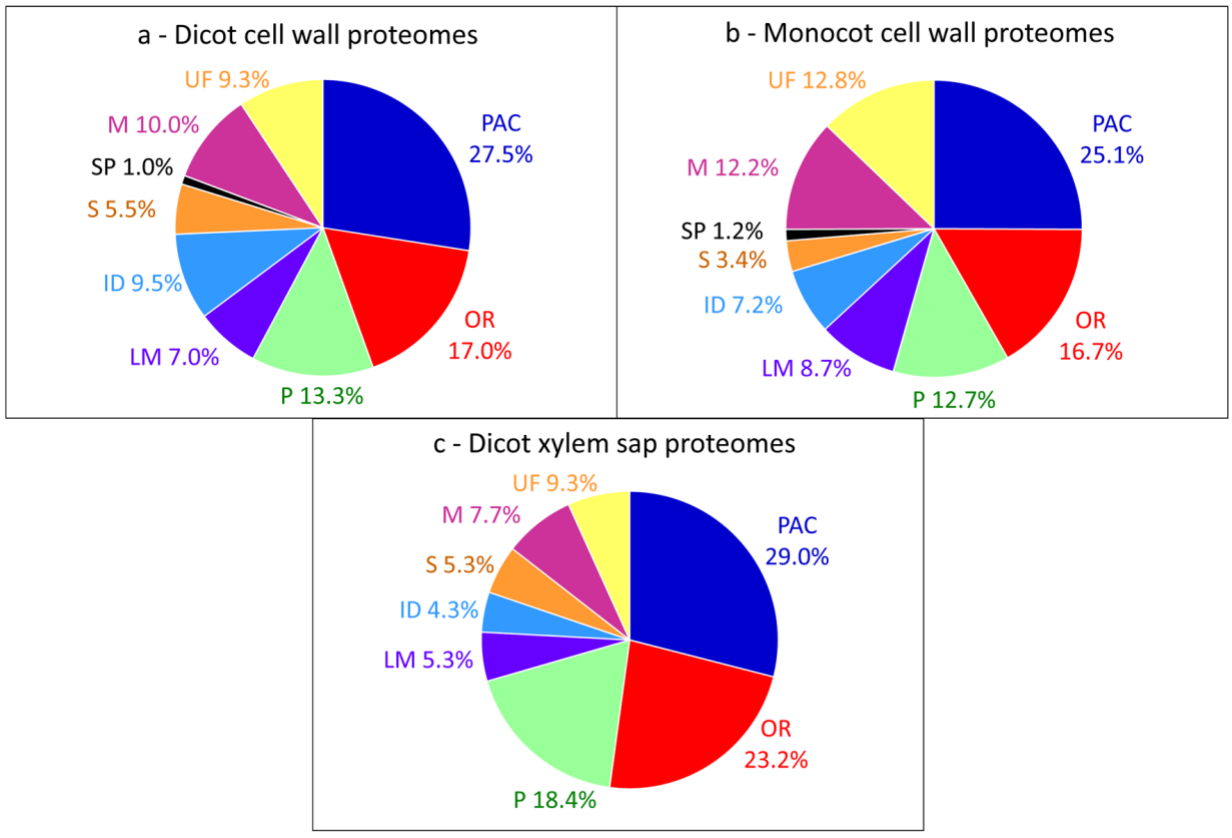
Distribution of CWPs into functional classes. All the proteins have been annotated according to the presence of functional domains (see Supplementary Material), thus providing homogeneous annotations. (**a**) Pool of dicot proteomes; (**b**) Pool of monocot proteomes; (**c**) Pool of xylem sap proteomes.

To date, the overall distribution of CWPs into these functional classes is similar between dicot and monocot cell wall proteomes with three major classes (Figure 1a,b): PAC (around 26%), oxido-reductases (around 17%), and proteases (around 13%). These average proteomes contain data (i) originating from different kinds of plant organs or from cell suspension cultures, (ii) obtained using various methods of extraction and (iii) identified using different mass spectrometry techniques [10]. They give an overview of the types of proteins which can be identified using the variety of available strategies. Although xylem sap proteomes contain CWPs [19,52], their distribution into functional classes is very different from that of CWPs extracted from plant organs (Figure 1c), with a higher proportion of PAC, oxido-reductases and proteases. 

**Figure 2:**
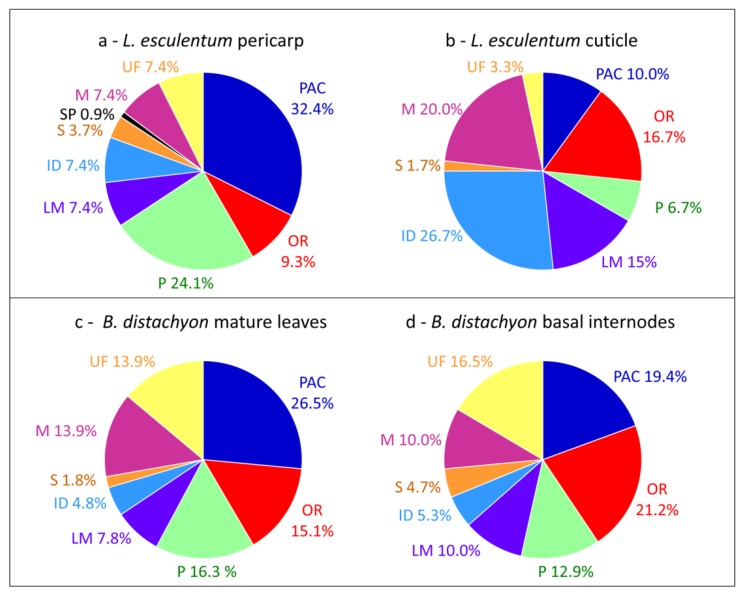
Comparisons of cell wall proteomes of different plant tissues or organs. (**a**) *L. esculentum* fruit pericarp; (**b**) *L. esculentum* fruit cuticle; (**c**) *B. distachyon* mature leaves; (**d**) *B. distachyon* basal internodes. All the proteins have been annotated according to the presence of functional domains (see Supplementary Material).

Interestingly, differences can be highlighted when comparisons of cell wall proteomes obtained in similar conditions are done between different tissues or organs of the same plant (Table 2). The comparison of the cell wall proteomes of *Solanum lycopersicum* fruit pericarp [20] and cuticle [53] shows striking changes in the relative importance of PAC (32.4% *vs.* 10.0%), oxido-reductases (9.3% *vs.* 16.7%), proteases (24.1% *vs.* 6.7%), proteins related to lipid metabolism (7.4% *vs.* 15%), proteins having interacting domains (7.4% *vs.* 26.7%) and miscellaneous proteins (7.4% *vs.* 20.0%) (Figure 2a,b). It is not surprising that the proportion of PAC is lower in the cuticle proteome than in the pericarp cell wall proteome and that the proportion of proteins related to lipid metabolism is higher. Indeed, the biogenesis of the cuticle composed of waxes and cutin occurs at the plant surface [53]. In the same way, major differences are found between cell wall proteomes of mature leaves and basal internodes of *Brachypodium distachyon* [15]: 26.5% *vs.* 19.4% PAC and 15.1% *vs.* 21.2% oxido-reductases (Figure 2c,d). Although both organs are mature, basal internodes are more lignified than mature leaves and the presence of more oxido-reductases and less PAC is probably required for lignin monomer polymerization.

**Table 2:** Information about the cell wall or xylem sap proteomes used for overall comparisons.

	Stems	Leaves	Fruit pericarp	Fruit cuticle	Xylem sap	Protocols	Ref.
**Dicots**							
*B. napus/oleracea*					x	xylem sap	[19]
*L. usitatissimum*	x					- cell wall preparation - extraction of proteins from cell walls with CaCl_2_, LiCl	[46]
*M. sativa*	x					- cell wall preparation - extraction of proteins from cell walls with EGTA, LiCl	[16]
*P. deltoides*					x	xylem sap	[13]
*S. lycopersicum*				x		chloroform extraction	[53]
*S. lycopersicum*			x			*N*-glycoproteome (total protein extraction followed by ConA affinity chromatography	[20]
*S. tuberosum*		x				- cell wall preparation - extraction of proteins from cell walls with CaCl_2_	[55]
**Monocots**							
*B. distachyon*	x	x				- cell wall preparation - extraction of proteins from cell walls with CaCl_2_, LiCl	[15]

**Figure 3:**
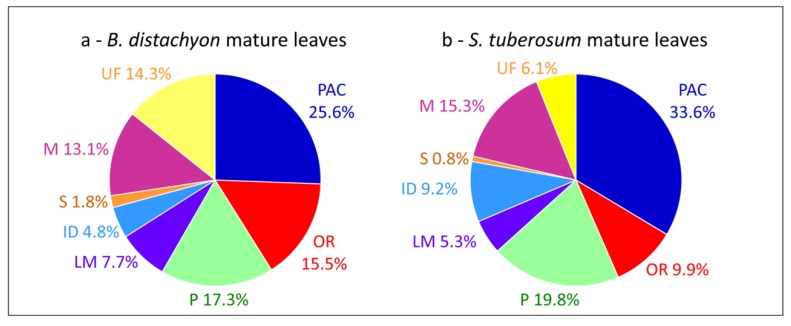
Comparisons of cell wall proteomes of mature leaves between a monocot and a dicot. (**a**) *B. distachyon*; (**b**) *S. tuberosum*. All the proteins have been annotated according to the presence of functional domains (see Supplementary Material).

Comparisons of cell wall proteomes between similar organs of monocots and dicots show differences related to the composition of their cell walls [1]. For example, cell wall proteomes of leaves of *B. distachyon* [15] and *Solanum tuberosum* [55] show differences in the relative proportions of PAC (25.6% *vs.* 33.6%), oxido-reductases (15.5% *vs.* 9.9%), proteins related to lipid metabolism (7.7% *vs.* 5.3%) and proteins having interacting domains (4.8% *vs.* 9.2%) (Figure 3a,b). In both cases, proteins have been extracted from purified cell walls using salt solutions. Such differences have been discussed [15]. It was suggested that the presence of aromatic compounds in monocot primary cell walls could explain the higher proportion of oxido-reductases. The higher proportion of proteins related to lipid metabolism has been related to the presence of a cuticle on both sides of monocot leaves. Finally, only a few enzyme inhibitors have been identified in the *B. distachyon* leaf proteome as well as no lectin. A similar comparison between cell wall proteomes of stems such as those of *B. distachyon* [15], *Linum usitatissimum* [46] and *Medicago sativa* [16] does not show striking differences between monocots and dicots probably because both contain lignified secondary walls.

All these comparisons are qualitative ones based on presence/absence of proteins in cell wall proteomes. Inside each functional class, the comparison of protein families can be refined to look for candidate proteins possibly involved in cell wall remodeling in specific organs, during particular stages of development, or in response to changes in environmental conditions. Such results are discussed in detail in experimental papers (see Table 1). Quantitative data are still scarce and the limitations of the available protocols to completely extract CWPs from cell walls do not allow getting fully reliable information as for transcriptomes. However, transcriptomic data do not provide any information about post-transcriptional levels of gene regulation, and both types of data are complementary [65]. 

## 3. The Limitations for Full Coverage of Cell Wall Proteomes

Although well-documented, plant cell wall proteomes are probably missing proteins lost during the purification of cell walls and important protein families such as structural proteins are still lacking. These limitations will be examined in the following paragraphs [30]. 

### 3.1. Loss of Proteins during the Purification of Cell Walls

It is difficult to obtain a high coverage of the complete set of proteins present in cell walls because of the lack of surrounding membrane which can result in the loss of CWPs during the isolation procedure [66]. CWPs can have little or no interactions with cell wall components and thus move freely in the extracellular space. Non-destructive techniques such as vacuum infiltration [25], or recovery of liquid culture media from cell suspension cultures or seedlings [23,27] were developed to overcome this obstacle. Large sets of “labile CWPs” have been identified. Most of them have acidic pI ranging from 2 to 6 while CWPs are mainly basic proteins [67].

**Figure 4:**
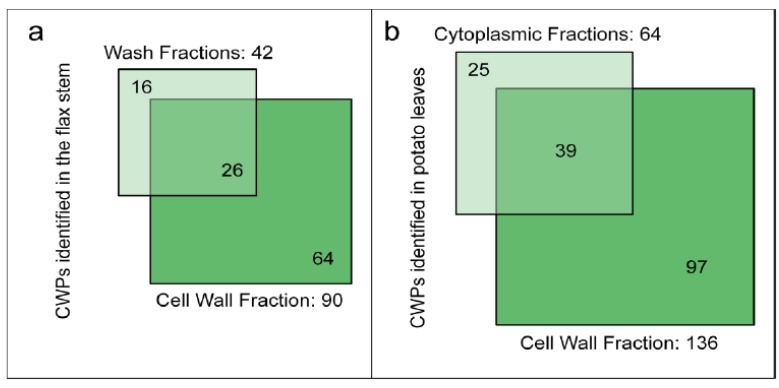
Diagrams indicating the number of identified flax or potato cell wall proteins in different fractions. (**a**) Wash and cell wall fractions from flax stem (data from [46]); (**b**) Cytoplasmic and cell wall fractions from potato leaves (data from [55]). The sub-cellular localization of proteins has been predicted as described in Supplementary Material. Only proteins having a predicted signal peptide and no known intracellular retention signal are considered as CWPs.

Two recent studies using destructive methods to isolate cell walls of flax stems or potato leaves have considered the loss of proteins during the cell wall purification steps [46,55]. Starting with ground plant material, the isolation procedures retained a differential centrifugation approach to separate cell wall and cytoplasmic fractions [55]. Several washing steps were performed to exclude cytoplasmic and membrane proteins [46]. Figure 4 shows the number of CWPs identified in the different fractions, *i.e.*, wash *vs.* cell wall fractions (flax stem) and cytoplasmic *vs.* cell wall fractions (potato leaves). Surprisingly, about 15% of the CWPs identified in these studies were only present in the wash or in the cytoplasmic fractions. These CWPs did not show any distinctive features, e.g., their pIs are in the basic range in contrast to the “labile CWPs” identified with non-destructive methods and no particular protein family could be found [67]. The isolation procedures used to purify cell walls led to a significant loss of CWPs. The wash and cytoplasmic fractions could also be considered in cell wall proteomic studies. However, in flax, while 958 proteins have been identified in the wash fraction, only 42 are predicted to be secreted (about 4%). The main drawback is the identification of a large number of intracellular proteins whereas CWPs are in the minority. 

### 3.2. Extraction of Proteins by Salt Solutions

Most plant cell wall proteomic studies use salts to release CWPs from cell walls using non-destructive strategies or to extract proteins from purified cell walls [10]. Different types of salt solutions have been used, but CaCl_2_ solutions appeared to be among of the most efficient ones [25]. In the case of destructive methods, there are doubts with regard to the release of *bona fide* CWPs since the intracellular content is released at the time of tissue grinding. Actually, two kinds of proteins are identified, those having predicted signal peptides which are considered as CWPs in this review, and those having no signal peptide. This point has been discussed in previous reviews [10,68]. 

To illustrate the efficiency of CWP extraction from purified call walls using salt solutions, we have examined the cell wall localization of a protein identified in numerous cell wall proteomic studies, namely At5g11420. This is one of the so-called DUF642 (Domain of Unknown function) proteins which all have a predicted signal peptide [69]. In addition, since the observation of fluorescent chimeric proteins by confocal microscopy offers the opportunity to explore the effect of exogenous treatments on the protein localization dynamic at the cellular scale, we show the release of At5g11420 after a salt solution treatment. 

The plant cell wall is an acidic compartment and the sub-cellular localization of protein of interest labeled with a fluorescent protein (FP) is challenging in a low pH environment. The spectral properties of Green FP (GFP) are influenced by pH, and the fluorescence of GFP variants (e.g., monomeric Enhanced GFP, mEGFP and Yellow FP, YFP) decreases at a pH below 6. In this study, we have chosen the tagRFP as a fluorescent reporter taking advantage of its low pKa (3.1) [70]. 

**Figure 5:**
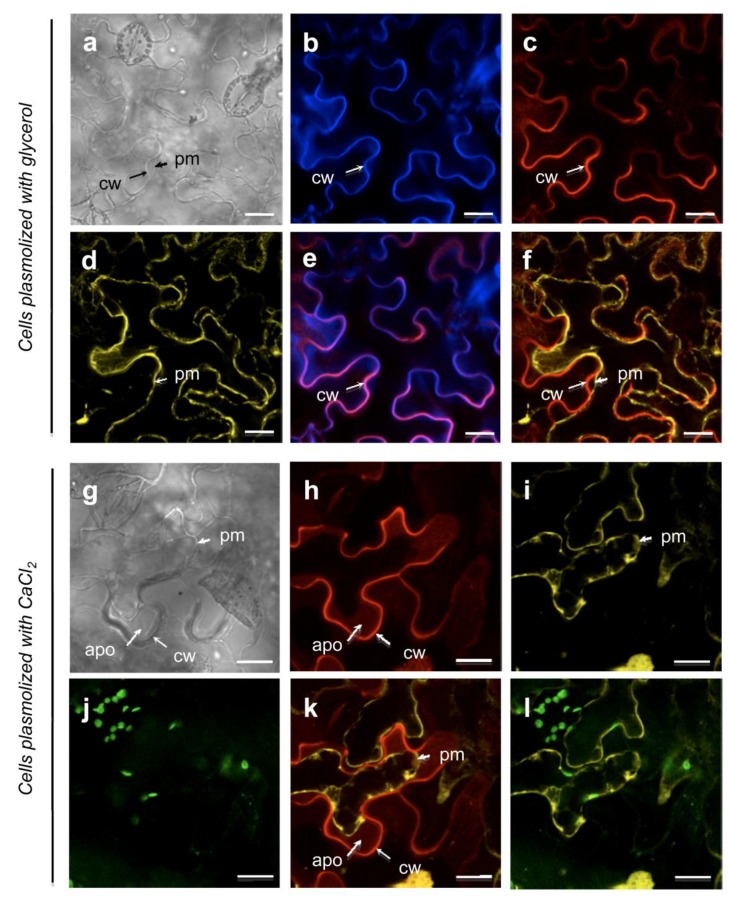
The At5g11420 protein is localized in the cell wall (see Supplementary Material for methods). (**a**–**f**): *N. benthamiana* leaf epidermal cells plasmolyzed by incubation with glycerol. Cell wall localization of the At5g11420::tagRFP protein; (**g**–**k**): *N. benthamiana* leaf epidermal cells plasmolyzed by incubation with CaCl_2_. Under CaCl_2_ treatment the At5g11420::tagRFP protein partially relocalizes to the apoplasm; (**a**, **g**) Bright field; (**b**) Calcofluor labelling of the cell wall; (**c**, **h**) RFP labelling; At5g11420::tagRFP was used to observe At5g11420 protein localization. (**d**, **i**) YFP labelling; aquaporin::YFP allows plasma membrane visualization; (**j**) Chloroplast labeling; (**e**) Merge of (**b**) and (**c**); (**f**, **k**) Merge of (**c**) and (**d**) and (**h**) and (**i**), respectively. (**l**) Merge of (**i**) and (**j**).

The *N. benthamiana* leaf epidermal cells, transiently expressing the p35S::At5g11420::tagRFP construct, produced a red fluorescent signal at the cell periphery (Figure 5c). In non-plasmolyzed and glycerol-plasmolyzed cells, the At5g11420::tagRFP protein co-localized with the calcofluor labelling, a specific cell wall marker (data not shown, Figure 5b,e). Under plasmolysis condition with glycerol, the plasma membrane labeled by the pm::YFP marker was progressively loosened from the cell wall, while the At5g11420::tagRFP fluorescence was maintained into the cell wall (Figure 5d,f). These data indicate that At5g11420 is specifically targeted to the cell wall.

When plasmolysis was induced by CaCl_2_, the detachment of the plasma membrane from the cell wall was accompanied by a new At5g11420::tagRFP labelling pattern (Figure 5h). After a few minutes of incubation, the At5g11420::tagRFP fluorescence diffused from the cell wall into the apoplastic compartment delimited by the plasma membrane (Figure 5k). This experiment illustrates how proteins can be released from cell walls using salt solutions. It should be noted that they can be released together with other cell wall components like pectins.

The efficiency of CWP extraction by salt solutions depends on the type of interactions between CWPs and cell wall components. This is also the reason why different extraction methods have been used in cell wall proteomic studies. Alternatively, glycoproteins have been captured by lectin affinity chromatography, starting from total extracts of proteins [20,37,44]. This strategy has proved to be very efficient since CWPs are synthesized in the secretory pathway. However, care should be taken to distinguish glycoproteins which are resident in the secretory pathway from those which are targeted to the extracellular space. 

cw, cell wall; pm, plasma membrane; apo, apoplasm. Bars = 20 µm.

### 3.3. Difficulties to Extract Structural Proteins

As mentioned above, cell wall proteomic studies mentioned in this review rely on protein extraction methods using salt extractions. However, these strategies were shown to be inefficient to solubilize covalently-linked proteins, like structural proteins. To date, only a few PRPs, Leucine-Rich repeat Extensin (LRXs), Glycine-Rich Proteins (GRPs) or Thr/Hyp-rich GlycoProteins (THRGPs) have been identified (Table 3). Structural proteins are thus under-represented in cell wall proteomes, *i.e*., 3 PRPs and no EXT out of the 18 and 32 respectively predicted in *A. thaliana* [71]. Main features concerning these families are reported below to pinpoint the bottlenecks preventing their extraction.

**Table 3:** Structural proteins identified in cell wall proteomes.

Protein family	Plant	References
**PRP** (At5g09530; At5g14920, AtGASA14)	*A. thaliana*	[14]
**AGP/PRP** (At1g28290, AtAGP31)	*A. thaliana*	[14,30]
**LRX** (At1g62440, AtLRX2; At4g13340; At3g24480; AtLRX3, AtLRX4; At4g18670, AtLRX5)	*A. thaliana*	[14,22,38]
**GRP** (At2g05580)	*A. thaliana*	[14]
**LRX** (Os01g0594300, Os05g0180300, Os06g0704500, Os02g0138000	*O. sativa*	[56]
**GRP** (Os07g0688700, Os07g0440100)	*O. sativa*	[57]
**THRGP** (Os03g0676300, Os04g0418800)	*O. sativa*	[56,57]
**AGP/PRP** (Lus10015434)	*L. usitatissimum*	[46]
**LRX** (Medtr8g103700.1, Medtr6g086120.1)	*M. sativa*	[16]
**LRX** (Solyc11g005150.1)	*L. esculentum*	[20]

EXTs belong to the superfamily of hydroxyproline-rich glycoproteins (HRGPs) and are involved in cell wall assembly, cell shape and growth [72,73,74]. They have been widely studied since the sixties and constitute one of the best known CWP family [75]: (i) they are basic proteins, (ii) they contain repetitive sequence with contiguous Hyp *O*-glycosylated with short arabino-oligosaccharides, (iii) they adopt a polyproline II helical structure, (iv) they can be crossed-linked through isodityrosine or di-isodityrosine links [76] and (v) they interact with pectins. The molecular bases of their insolubilization have been highlighted recently. It was shown by atomic force microscopy (AFM) analysis that the purified *A. thaliana* AtEXT3 self-assemble to form dendritic structures, consistent with cross-linking by peroxidases observed *in vitro* [77]. Similar network structures were observed by AFM for a maize THRGP, but peroxidases were not involved in their cross-linking [78]. AFM observations corroborate previously reported electronic microscopy data showing intramolecular and short intermolecular cross-links [79]. It was proposed that self-assembled extensins form positively charged scaffolds in the cell plate, able to react with negatively charged pectins through ionic interactions. Besides, covalent cross-links between extensins and pectins were also suggested [80,81]. 

EXT-like chimeras and hybrid-EXTs also exist in cell walls [72,73]. They are assumed to be insolubilized *in muro* but the presence of other protein domains may modify their behavior. For instance, the *A. thaliana* LRX1 is insolubilized in the cell wall, but this does not involve Tyr cross-links [82]. However, Tyr residues are required for LRX1 function in root hair formation [82]. 

PRPs are highly basic, mostly lowly glycosylated proteins, and they display specific repetitive motifs [83,84]. PRPs are probably covalently cross-linked in the cell wall, but direct evidence is still lacking [85,86,87]. 

GRPs are characterized by a high content in glycine residues (up to 70%) [88,89]. Several studies using immunocytochemistry have shown that they are associated with the protoxylem, suggesting a function in a repair system during the stretching phase [88]. It is assumed that the repetitive nature of the glycine-rich domains leads to the formation of β-pleated sheet structures allowing hydrophobic interactions. Interestingly, *in vitro* cross-linking experiments carried out in presence of peroxidase suggested the formation of networks only in Tyr-containing GRPs [90]. However, further experimental data should be obtained to characterize with more details intra- and inter-molecular networks involving GRPs *in muro.*

Finally, some AGPs were shown to bind covalently to the cell wall. They constitute a category of HRGPs *O*-hyperglycosylated by arabinogalactans at non-contiguous Hyp, playing essential roles in a wide range of plant growth and development processes [91]. AGPs have been assumed to form complexes with pectins and xylans [91]. The first experimental evidence for covalent attachment between an *A. thaliana* AGP and hemicellulosic and pectic polysaccharides, forming a complex called Arabinoxylan Pectin Arabinogalactan Protein1 (APAP1), has been recently reported [92]. Interestingly, the *apap1* mutant showed an increased extractability of pectin and xylan, supporting the structural role proposed for APAP1 [92]. This result indicates that some AGPs may serve as cross-linker in cell walls, corroborating previous reports where AGPs were described as pectic plasticizers [93,94]. 

Alternative extraction strategies using SDS buffer to extract structural proteins have been tried but they were inefficient [30]. The question of the extraction of covalently bound CWPs thus remains unanswered and further research is necessary to improve their identification by proteomics.

## 4. Concluding Remarks

The knowledge of plant cell wall proteomes has been greatly enlarged through the numerous studies performed during the last fifteen years. Thanks to various complementary strategies, it is possible to get an overview of proteins present in the cell walls of numerous plant organs and in cell suspension cultures. However, the full coverage of plant cell wall proteomes remains challenging since some proteins are lost during the purification of cell walls and cross-linked proteins are not extracted. Global approaches avoiding cell wall purification such as direct capture of glycoproteins on lectin affinity columns did not allow to significantly enlarge cell wall proteomes [20,37,44]. It can be anticipated that a better coverage of cell wall proteomes will require strategies adapted to protein families of interest as for AGPs which have been specifically targeted by the Yariv reagent [35]. 

A major drawback for the use of cell wall proteomic data is the heterogeneity of protein functional annotation which limits relevant interpretation of data and comparisons between proteomes [95]. In this regard, WallProtDB is a useful tool since all the proteins are annotated in the same way. At present, it is probable that the identified proteins are the most abundant and the most accessible within the intricate extracellular polysaccharide networks. Besides, reliable quantitative information is now required to better describe CWP profiles and correlate them to plant physiological state.

## Supplementary Materials

Supplementary File 1
