# Supplementary material for: Plant Cell Wall Proteins: A Large Body of Data, but What about Runaways?
Source: Proteomes. 2014 Apr 17;2(2):224–42. doi: 10.3390/proteomes2020224 (PMC5302738; doi:10.3390/proteomes2020224)
Supplement: Supplementary File 1 [file proteomes-02-00224-s001.pdf]

Supplementary Material for

Plant Cell Wall Proteins: A Large Body of Data, but What about Runaways?

1. Bioinformatics

All the proteins considered in this review have been re-annotated using the ProtAnnDB pipeline to allow a comparison between the published proteomes based on the same prediction of sub-cellular localization and of functional domains [1,2]. Only proteins predicted to have a signal peptide with at least two different bioinformatic programs were retained as CWPs. Most of the cell wall proteomes discussed in this paper are included in WallProtDB [3].

2. Methods for Sub-Cellular Localization of At5g11420

The At5g11420::tagRFP construct has been generated by using the Gateway recombination technology (Invitrogen^{TM}, Life Technologies SAS, Saint Aubin, France). At first, the attB1::At5g11420::tagRFP::attB2 PCR product has been obtained as following. The pBAD-TOPO vector containing the *At5g11420* coding sequence has been used as template to perform a PCR with the sense and antisense primers 5'-aaaaagcaggcttcatgggctctggatccgg-3' and 5'-acctccgccggatccaccgccg gaatggtgatggtgatgatgacc-3', respectively. The tagRFP coding sequence has been amplified from the pTagRFP-C vector (FP141, Evrogen, Euromedex, Souffelweyersheim, France) with the sense and antisense primers 5'-tccggcggtggatccggcggaggtatggtgtctaagggcgaagagctga-3' and 5'-agaaagctgggtcctaa ttaagtttgtgccccagtttgcta-3', respectively. The PCR products have been fused by the overlap extension PCR method, and the corresponding fused DNA product attB1::At5g11420::tagRFP::attB2 has been amplified with the sense and antisense primers 5'-ggggacaagtttgtacaaaaaagcaggct-3' and 5'-ggggaccactttgtacaagaaagctgggt-3', respectively. Finally, the attB1::At5g11420::tagRFP::attB2 PCR product has been cloned into the pDONR207 by BP reaction, and then, into the pEAQ-*HT*-Dest1 destination vector by LR reaction [4]. The corresponding recombinant binary vector, with the sequence of interest At5g11420::tagRFP under the control of the 35S CaMV promoter is mentioned as p35S::At5g11420::tagRFP. All gateway recombination steps were performed according to the manufacturer's instructions (Invitrogen^{TM}).

Nicotiana benthamiana were grown in a growth room maintained at 25–22 °C, 80% of humidity and with 16 h of light. For transient co-localization studies, the plasma membrane aquaporin::YFP (p35S::pm::YFP) fusion protein was used as a specific fluorescent reporter protein for the plasma membrane (binary vector reference, pm-yb CD3-1006; [5]). p35S::At5g11420::tagRFP and p35S:pm:YFP binary vectors were maintained in *Agrobacterium tumefaciens* strain GV3101, which was transformed by electroporation. *N. benthamiana* leaves were infiltrated as described [6]. After 5 days, infected leaves were analyzed by confocal microscopy.

Observations were performed with a Leica TCS SP2 AOBS confocal laser-scanning microscope. Infected leaves were stained with 0.1% calcofluor for 15 min. Cell plasmolysis was performed with either 30% glycerol or 0.5 M calcium chloride. YFP was excited at 514 nm and detected in the 521–550 nm range. TagRFP was excited at 561 nm and detected in the 568–614 nm range. Calcofluor

was excited at 405 nm and detected in the 417–488 nm range. Chlorophyll was excited at 633 nm and detected in the 654–678 nm range. No image enhancement was performed.

References

1. San Clemente, H.; Pont-Lezica, R.; Jamet, E. Bioinformatics as a tool for assessing the quality of sub-cellular proteomic strategies and inferring functions of proteins: Plant cell wall proteomics as a test case. *Bioinform. Biol. Insights* **2009**, *3*, 15–28.
2. ProtAnnDB. Available online: http://www.polebio.lrsv.ups-tlse.fr/ProtAnnDB/ (accessed on 15 January 2014).
3. WallProtDB. Available online: http://www.polebio.lrsv.ups-tlse.fr/WallProtDB/ (accessed on 20 March 2014).
4. Sainsbury, F.; Thuenemann, E.; Lomonossoff, G. pEAQ: Versatile expression vectors for easy and quick transient expression of heterologous proteins in plants. *Plant Biotechnol. J.* **2009**, *7*, 682–693
5. Nelson, B.; Cai, X.; Nebenführ, A. A multicolored set of *in vivo* organelle markers for co-localization studies in *Arabidopsis* and other plants. *Plant J.* **2007**, *51*, 1126–1136.
6. Voinnet, O.; Rivas, S.; Mestre, P.; Baulcombe, D. An enhanced transient expression system in plants based on suppression of gene silencing by the p19 protein of tomato bushy stunt virus. *Plant J.* **2003**, *33*, 949–956.

© 2014 by the authors; licensee MDPI, Basel, Switzerland. This article is an open access article distributed under the terms and conditions of the Creative Commons Attribution license (http://creativecommons.org/licenses/by/3.0/).
